# Supplementary material for: Comparison of Nutrition Indices for Prognostic Utility in Patients with Sepsis: A Real-World Observational Study
Source: Diagnostics (Basel). 2023 Mar 30;13(7):1302. doi: 10.3390/diagnostics13071302 (PMC10093319; doi:10.3390/diagnostics13071302)

Supplementary Figure S1 Non-linear cubic spline curve of nutrition indexes against mortality in sepsis between non-ICU group and ICU group.

Supple. S1A GNRI (left: non-ICU, right: ICU)

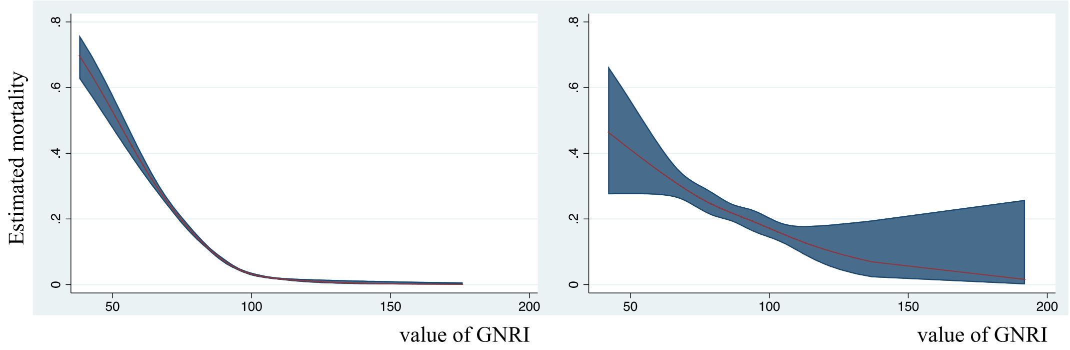

Supple. S1B PNI (left: non-ICU, right: ICU)

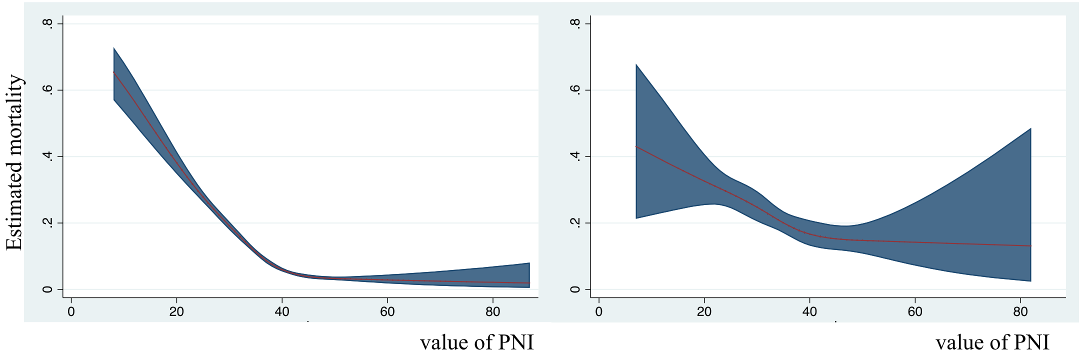

Supple. S1C CONUT (left: non-ICU, right: ICU)

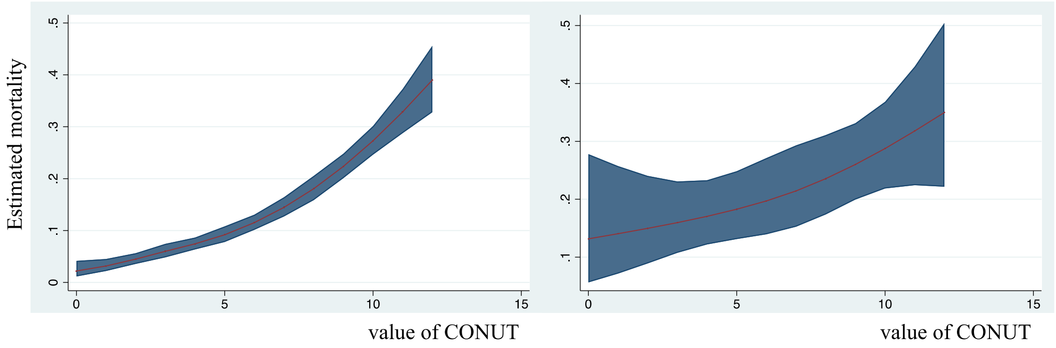

Supplement: Supplementary file 1 [file diagnostics-13-01302-s001.zip › diagnostics-2204439-supplementary.pdf]
